# Supplementary material for: Resolving the full spectrum of human genome variation using Linked-Reads
Source: Genome Res. 2019 Apr;29(4):635–45. doi: 10.1101/gr.234443.118 (PMC6442396; doi:10.1101/gr.234443.118)
Supplement: Supplemental Material [file supp_gr.234443.118_Supplemental_longranger-2.2.zip › longranger-2.2.tar.bz2!/lr2.2-public/lib/stan/cmdstan/stan/doc/api/html/functions_func_m.html]

Stan: Class Members - Functions


|  |  |
| --- | --- |
|  | Stan  2.5.0  probability, sampling & optimization |


- Main Page
- Related Pages
- Namespaces
- Classes
- Files

- Class List
- Class Index
- Class Hierarchy
- Class Members

- All
- Functions
- Variables
- Typedefs
- Enumerator
- Related Functions

- a
- b
- c
- d
- e
- f
- g
- h
- i
- j
- k
- l
- m
- n
- o
- p
- q
- r
- s
- t
- u
- v
- w
- x
- z
- ~


All Classes Namespaces Files Functions Variables Typedefs Enumerations Enumerator Friends Macros Pages

### - m -

- matrix()
  : stan::io::reader< T >
- matrix\_constrain()
  : stan::io::reader< T >
- matrix\_lb()
  : stan::io::reader< T >
- matrix\_lb\_constrain()
  : stan::io::reader< T >
- matrix\_lb\_unconstrain()
  : stan::io::writer< T >
- matrix\_lub()
  : stan::io::reader< T >
- matrix\_lub\_constrain()
  : stan::io::reader< T >
- matrix\_lub\_unconstrain()
  : stan::io::writer< T >
- matrix\_ub()
  : stan::io::reader< T >
- matrix\_ub\_constrain()
  : stan::io::reader< T >
- matrix\_ub\_unconstrain()
  : stan::io::writer< T >
- matrix\_unconstrain()
  : stan::io::writer< T >
- matrix\_var\_decl()
  : stan::gm::matrix\_var\_decl
- matrixLDLT()
  : stan::math::LDLT\_factor< T, R, C >
- max()
  : std::numeric\_limits< stan::agrad::fvar< T > >
  , std::numeric\_limits< stan::agrad::var >
- mcmc\_writer()
  : stan::io::mcmc\_writer< M, SampleRecorder, DiagnosticRecorder, MessageRecorder >
- mean()
  : stan::mcmc::chains< RNG >
- member\_var\_decl\_visgen()
  : stan::gm::member\_var\_decl\_visgen
- messages()
  : stan::common::recorder::messages
- min()
  : std::numeric\_limits< stan::agrad::fvar< T > >
  , std::numeric\_limits< stan::agrad::var >
- minimize()
  : stan::optimization::BFGSMinimizer< FunctorType, QNUpdateType, Scalar, DimAtCompile >
- model\_functional()
  : stan::model::model\_functional< M >
- ModelAdaptor()
  : stan::optimization::ModelAdaptor< M >


---

[ Stan Home Page ]

*© 2011–2014,
Stan Development Team.*
